# Fabrication and Characterization of In_0.53_Ga_0.47_As/InAs/In_0.53_Ga_0.47_As Composite Channel Metamorphic HEMTs (mHEMTs) on a GaAs Substrate

**DOI:** 10.3390/mi14010056

**Published:** 2022-12-25

**Authors:** Seung Heon Shin, Jae-Phil Shim, Hyunchul Jang, Jae-Hyung Jang

**Affiliations:** 1 Department of Semiconductor Process Equipment, Semiconductor Convergence Campus, Korea Polytechnics, 41-12, Songwon-gil, Gongdo-eup, Anseong-si 17550, Republic of Korea; 2 Device Technology Division, Korea Advanced Nano Fab Center (KANC), 109, Gwanggyo-ro, Yeongtong-gu, Suwon-si 16229, Republic of Korea; 3 Department of Energy Engineering, Korea Institute of Energy Technology, 200, Hyeoksin-ro, Naju-si 58330, Republic of Korea

**Keywords:** mHEMT, HEMT, InAs HEMT, InGaAs HEMT, Mo-based Ohmic contact, In_0.53_Ga_0.47_As/InAs/In_0.53_Ga_0.47_As composite channel, GaAs, InGaAs/InAs/InGaAs composite channel

## Abstract

In this work, we successfully demonstrated In_0.53_Ga_0.47_As/InAs/In_0.53_Ga_0.47_As composite channel metamorphic high electron mobility transistors (mHEMTs) on a GaAs substrate. The fabricated mHEMTs with a 100 nm gate length exhibited excellent DC and logic characteristics such as *V_T_* = −0.13 V, *g_m,max_* = 949 mS/mm, subthreshold swing (SS) = 84 mV/dec, drain-induced barrier lowering (DIBL) = 89 mV/V, and I_on_/I_off_ ratio = 9.8 × 10^3^ at a drain-source voltage (*V_DS_*) = 0.5 V. In addition, the device exhibited excellent high-frequency characteristics, such as *f_T_/f_max_* = 261/304 GHz for the measured result and well-matched modeled *f_T_/f_max_* = 258/309 GHz at *V_DS_* = 0.5 V, which is less power consumption compared to other material systems. These high-frequency characteristics are a well-balanced demonstration of *f_T_* and *f_max_* in the mHEMT structure on a GaAs substrate.

## 1. Introduction

High electron mobility transistors (HEMTs) based on indium-rich In_x_Ga_1-x_As channel materials on an InP substrate have demonstrated excellent high-frequency and logic characteristics. In terms of high-frequency characteristics of the InGaAs channel HEMT, H. -B. Jo et al. demonstrated 738 GHz unity current gain cutoff frequency (*f_T_*) in a 19 nm In_0.8_Ga_0.2_As composite-channel HEMT on a InP substrate [1], D. -H. Kim et al. showed excellent logic performance and a *f_T_* of 644 GHz in 30 nm InAs Pseudomorphic HEMTs (pHEMTs) [2], and Northrop Grumman Corporation exhibited an *f_T_* of 610 GHz/*f_max_* of 1.5 THz by using an In_0.53_GaAs/InAs/In_0.53_GaAs composite channel with a L_g_ of 25 nm [3]. These remarkable performances have been achieved through downscaling of device feature size, an optimized fabrication process, and optimized InGaAs channel materials for excellent transport properties. In addition, InGaAs channel MOSFETs have shown outstanding logic performance on various substrates, such as InP and flexible substrates, with extensive efforts to enhance their capability in new device structures, S/D Ohmic contacts, and optimization of the gate stack [4,5,6,7]. Meanwhile, large-size and cheaper-cost substrates will be essential for large-volume manufacturing from a mass production point of view, but an InP substrate is more expensive than a GaAs substrate, and the size to date is limited to 6 inches. To overcome these limitations of the InP substrate, many groups have demonstrated many outstanding results for mHEMTs on a GaAs substrate [8,9,10,11]. In particular, Teledyne demonstrated excellent results of a 688 GHz *f_T_* by utilizing an In_0.7_GaAs mHEMT structure with dual Si δ-doping and an InAs-rich In_0.7_Al_0.3_As spacer on a GaAs substrate in 2011 [9]. Fraunhofer showed a maximum oscillation frequency (*f_max_*) exceeding 1000 GHz by using an In_0.8_GaAs mHEMT structure on a GaAs substrate in 2013 [11]. Among various HEMT structures, an InGaAs/InAs/InGaAs composite channel was used to enhance high-frequency characteristics in HEMT structures because of its excellent electron transport properties, such as electron velocity and mobility [2,3,12]. However, an In_0.53_Ga_0.47_As/InAs/In_0.53_Ga_0.47_As composite channel structure on a GaAs substrate has not been demonstrated yet. In this work, we fabricated an In_0.53_Ga_0.47_As/InAs/In_0.53_Ga_0.47_As composite channel HEMT on a GaAs substrate incorporating a molybdenum (Mo)-based Ohmic contact using blanket Mo deposition and investigated its electrical performance, such as DC, logic, and RF characteristics, with an L_g_ of 100 nm.

## 2. Layer Structure and Experiments

**Figure 1:**
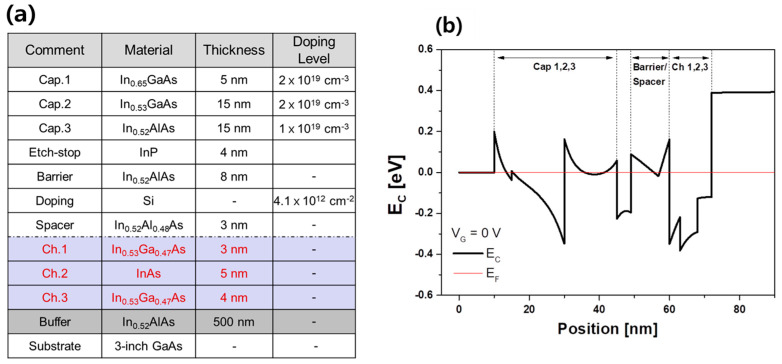

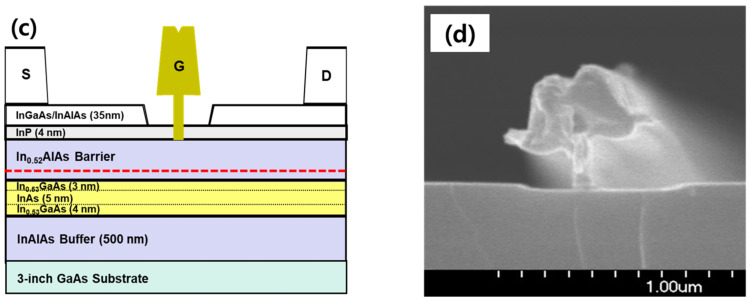
(**a**) Epitaxial structure of the In_0.53_Ga_0.47_As/InAs/In_0.53_Ga_0.47_As composite channel HEMT structure on a GaAs substrate. (**b**) Energy band diagram of the epitaxial structure (**c**) Schematic of the In_0.53_Ga_0.47_As/InAs/In_0.53_Ga_0.47_As composite channel HEMT on a GaAs substrate (**d**) SEM image of the fabricated T-gate.

The mHEMT heterostructures consisted of a 500 nm In_0.52_Al_0.48_As buffer, a 12 nm In_0.53_Ga_0.47_As/InAs/In_0.53_Ga_0.47_As (4/5/3 nm) channel, a 3 nm In_0.52_Al_0.48_As spacer, Si δ-doping (4.1 × 10^12^ cm^−2^), an 8 nm In_0.52_Al_0.48_As barrier, a 4 nm InP etch stop layer, and a 35 nm heavily doped In_0.53_Ga_0.47_As/In_0.52_Al_0.48_As multi-layer cap from the bottom to the top as shown in Figure 1a. The energy band diagram of the epitaxial structure is shown in Figure 1b. From this structure, sheet carrier density and electron hall mobility were measured to be 2.92 × 10^12^ cm^−2^ and 10,000 cm^2^/V·s at room temperature, respectively, with four-point probe measurement methods (Van der Pauw measurement method). Device fabrication began with a 30 nm blanket molybdenum (Mo) deposition for ohmic contact to prevent surface contamination and improve the contact resistance (R_c_), then mesa isolation down to an InAlAs buffer layer by Mo dry etching and wet etching. After Ti/Au/Ni (20/150/30 nm) metallization for source and drain, dry etching in an SF_6_/Ar plasma was performed to etch Mo in the gate region using the Ni metal etch mask of the source and drain [13]. A 30 nm thick layer of SiO_2_ was deposited by plasma-enhanced chemical vapor deposition (PECVD), and then the pad patterns with Ti/Au (20/300 nm) were defined for ground-signal-ground probing. After e-beam exposure, the defined e-beam resist pattern was transferred to define the T-gate by using reactive ion etching based on CF_4_ plasma. Gate recessing was performed in two different step stages, followed by anisotropic reactive ion etching of the InP etch stop layer in an Ar-based plasma [14]. After InP etching, Schottky gate metallization of Ti/Pt/Au (20/30/300 nm) was deposited on top of the InAlAs layer. Finally, the mHEMT with a width of 2 × 50 µm was fabricated, and a schematic of the fabricated mHEMT is shown in Figure 1c. Figure 1d shows the SEM image of the fabricated t-gate, whose foot and head sizes are 100 nm and 470 nm, respectively.

## 3. Results and Discussion

**Figure 2:**
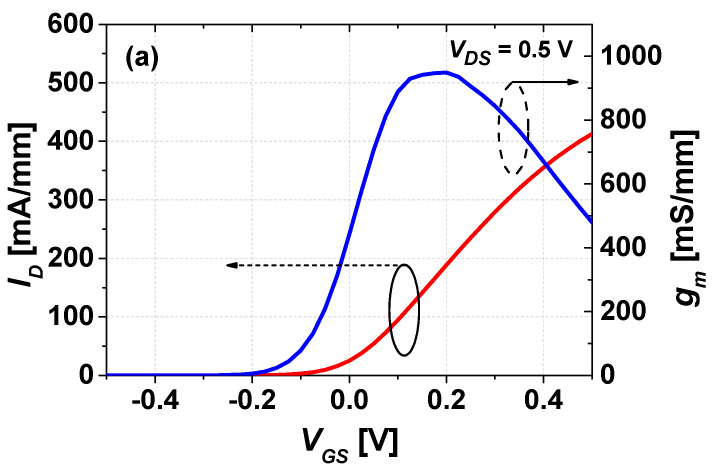

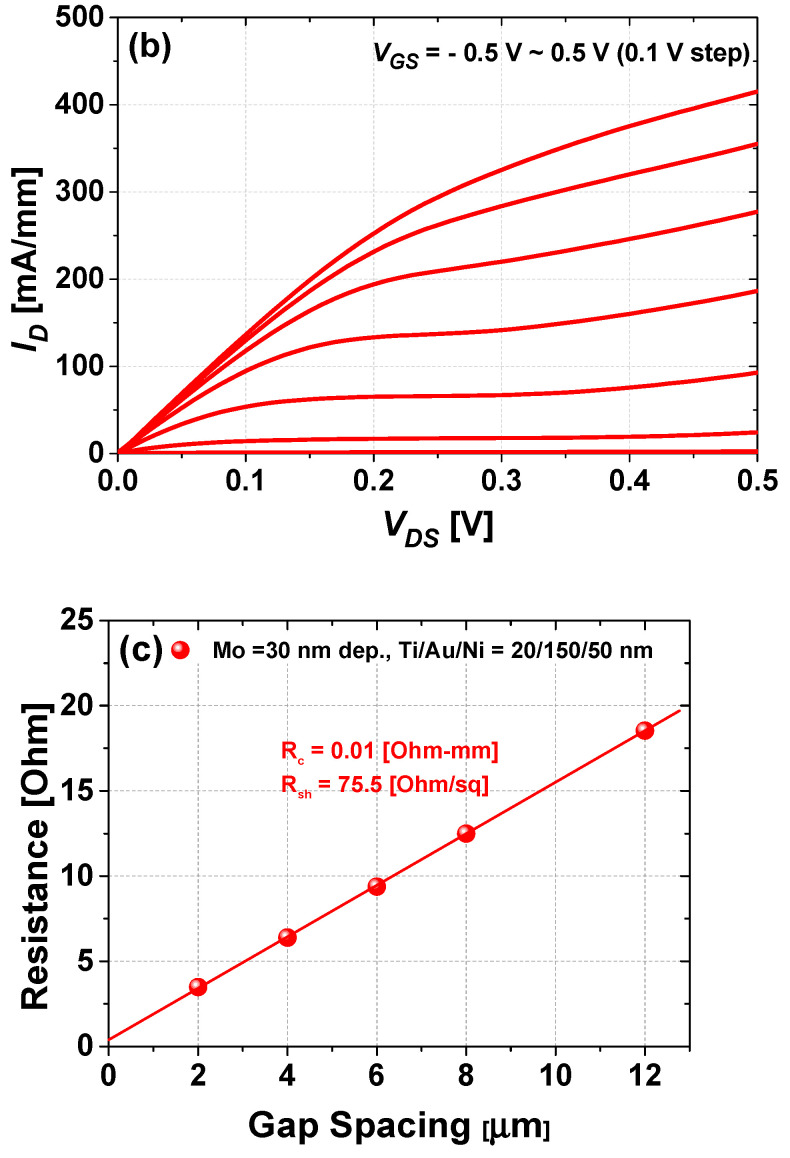
(**a**) Typical transfer characteristics of the mHEMTs measured at *V_DS_* = 0.5 V. (**b**) Typical output characteristics of the mHEMTs (*V_GS_* = −0.5 V ~ 0.5 V). (**c**) TLM result of the Mo-based Ohmic contact.

The transfer characteristic and output characteristic of the mHEMTs are shown in Figure 2. The maximum transconductance (*g_m,max_*) and maximum drain current density (*I_D,max_*) were 949 mS/mm and 413 mA/mm at *V_DS_* = 0.5 V, respectively. The output characteristics presented in Figure 2b show good pinch-off characteristics, but the measured R_on_ was 733 Ω-μm, which is a higher value than the lift-off Mo/Ti/Mo/Au metal scheme with an InAs rich InAlAs barrier spacer [15]. The ohmic contact resistance (R_c_) and sheet resistance (R_sh_) measured by the transmission line method (TLM) was as low as 0.01 Ω-mm and 75.5 Ω/sq as shown in Figure 2c. When compared to the lift-off method using a Mo/Ti//Mo/Au scheme (30/20/20/150 nm) on the same multi-cap layer, the blanket Mo method shows a higher R_sh_ value of 75.5 Ω/sq than the lift-off method of 69.2 Ω/sq because of SF_6_/Ar plasma damage in the active region during Mo etching to define the active gate region. However, the blanket Mo method shows a Rc of 0.011 Ohm-mm, which is lower than that of 0.026 Ohm-mm with the lift-off method because it is beneficial to protect the surface underneath the metal contact region from contaminants during the device process. Due to the lower R_c_ of the Mo blanket method, a lower R_on_ value could be achieved if the S/D distance was reduced, as in the self-aligned gate scheme.

**Figure 3:**
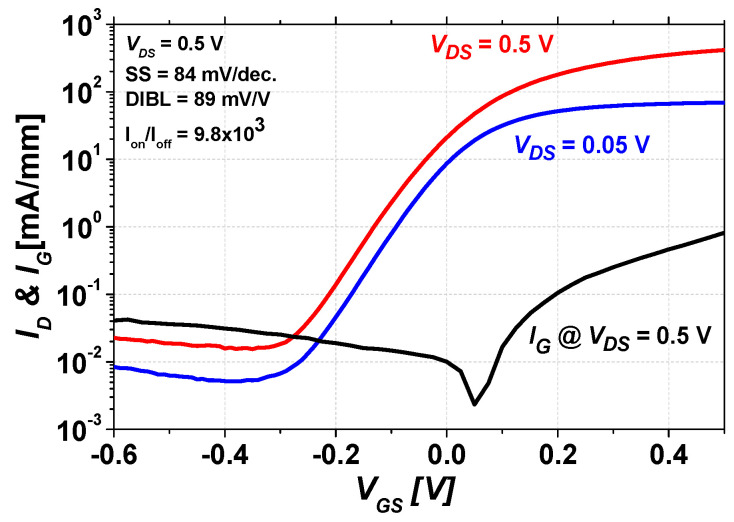
Subthreshold characteristics of the mHEMT were measured at *V_DS_* = 0.5 V and *V_DS_* = 0.05 V, and the gate leakage current was measured at *V_DS_* = 0.5 V, respectively.

Figure 3 shows the subthreshold characteristics at *V_DS_* = 0.5 V and 0.05 V, respectively. At *V_DS_* = 0.5 V, the threshold voltage (*V_T_*) is −0.13 V, defined as the value of *V_GS_* that yields at *I_D_* = 1 mA/mm, and a *V_T_* of −0.13 V indicates that the fabricated mHEMT operated in depletion mode (D-mode). The fabricated device shows excellent electrostatic integrity, such as the subthreshold swing (SS) of 84 mV/dec, the drain-induced barrier lowering (DIBL) of 89 mV/V, and the I_on_/I_off_ ratio of 9.8 × 10^3^, respectively. Additionally, the gate leakage current of the fabricated mHEMT was measured at *V_DS_* = 0.5 V and shows that the gate Schottky metallization is in good contact with the In_0.52_Al_0.48_As barrier layer. These outstanding logic performances are due to the well-designed heterostructure and optimized fabrication process on the GaAs substrate.

**Figure 4:**
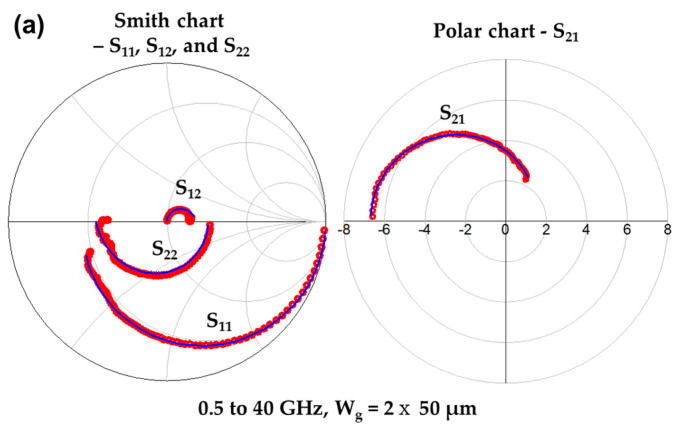

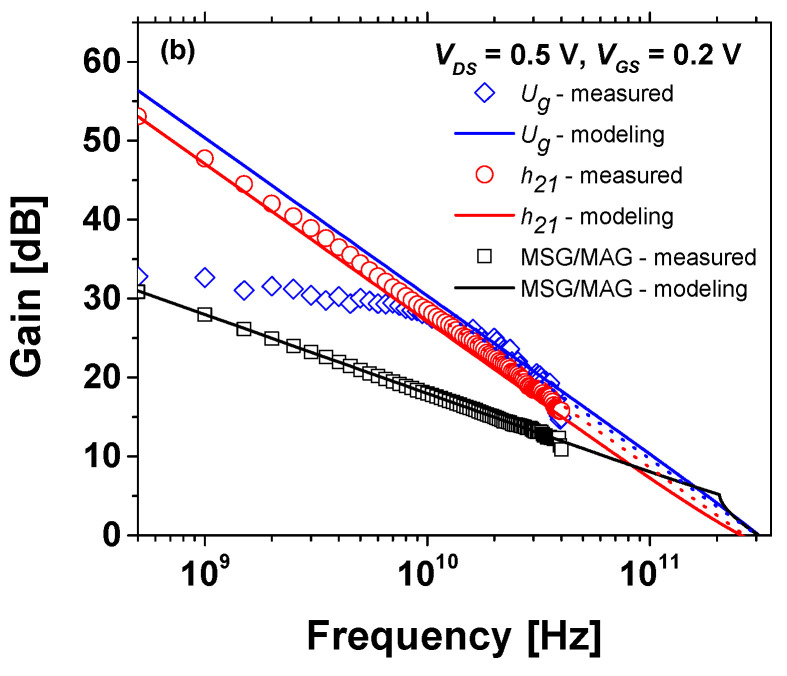
(**a**) Comparison of small-signal modeling and measured S-parameters at *V_DS_* = 0.5 V and *V_GS_* = 0.2 V. A Smith chart of S_11_, S_12_, and S_22_ (left) and a polar chart of S_12_ (right). (**b**) Measured (symbols) and modeled (solid lines) of RF gains-Maximum oscillation frequency (*f_max_*), maximum stable gain (MSG)/maximum available gain (MAG), and unity current gain cutoff frequency (*f_T_*) of the mHEMTs at *V_DS_* = 0.5 V and *V_GS_* = 0.2 V.

To verify the high-frequency RF characteristics of the mHEMT, S-parameters were measured from 0.5 to 40 GHz using a vector network analyzer (VNA). In addition, small-signal modeling was performed by using a small-signal equivalent circuit [16], and we found that small-signal modeling and measured S-parameters are well matched, as shown in Figure 4a. Figure 4b shows the unity current gain cutoff frequency (*f_T_*), maximum oscillation frequency (*f_max_*), and maximum stable gain (MSG)/maximum available gain (MAG) against frequency for the measured results (symbols) and modeled results (solid lines) at *V_DS_* = 0.5 V and *V_GS_* = 0.2 V with a *L_g_* of 100 nm mHEMT device. The de-embedding method was done by using open and short patterns to extract parasitic pad capacitance and inductance. We obtained 261 GHz/304 GHz for *f_T_*/*f_max_* by extrapolation (dashed lines) and 258 GHz/309 GHz for *f_T_*/*f_max_* by small-signal modeling, respectively. This excellent high-frequency response is due to the high value of the intrinsic transconductance (*g_mi_*) of 2.0 mS/µm. The extracted intrinsic parameters of the mHEMT are summarized in Table 1 and are well-matched to the measured results. The difference between *g_m,ext_* (0.95 mS/µm) and *g_mi_* (2.0 mS/µm) is due to the *R_s_* and *g_o_* values according to equation (1) [17].

(1) $$g_{m,ext}=g_{mi}\left(1-2R_{s}\cdot g_{o}\right)/\left(1+R_{s}\cdot g_{mi}\right)$$

**Figure 5:**
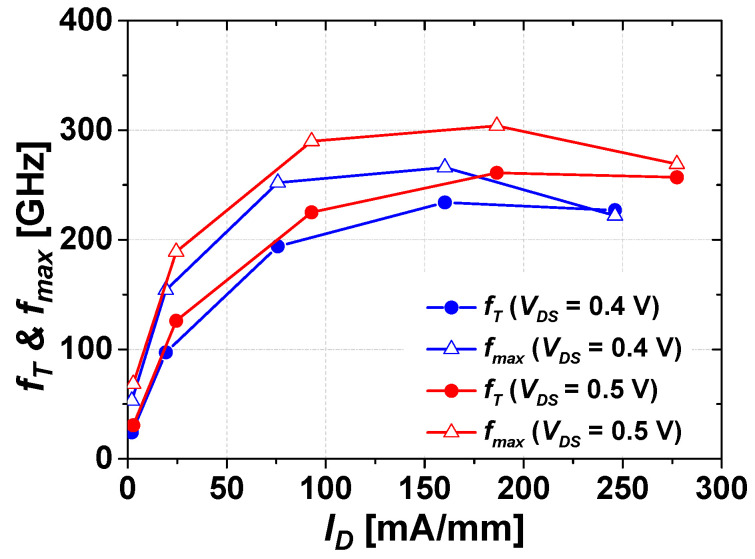
Maximum oscillation frequency (*f_max_*) and unity current gain cutoff frequency (*f_T_*) of the mHEMTs against drain current density (*I_D_*) at a *V_DS_* = 0.5 and 0.4 V, respectively.

Figure 5 shows *f_T_* and *f_max_* as functions of *I_D_* at *V_DS_* = 0.5 V and 0.4 V. Around an *I_D_* of 75 mA/mm, our device had already exhibited an *f_T_* and *f_max_* value of over 200 GHz and was confirmed to operate stably for the fabricated mHEMT.

Table 2 shows the benchmark high-frequency characteristics of the published state-of-the-art pHEMT and mHEMT results with an *L_g_* of 100 nm. Among various HEMT structures, the In_0.53_Ga_0.47_As/InAs/In_0.53_Ga_0.47_As composite channel HEMT on an InP substrate shows excellent high-frequency characteristics such as an *f_T_* of 421 GHz and an *f_max_* of 620 GHz because of the well-optimized fabrication process and improved carrier transport properties of the In_0.53_Ga_0.47_As/InAs/In_0.53_Ga_0.47_As composite channel [18]. Our fabricated mHEMT exhibits an excellent *L_g_f_T_* of 26.1 GHz-µm, which is related to carrier transport properties [19], and an outstanding *f_T_/f_max_* of 261/304 GHz with a *L_g_* of 100 nm at a *V_DS_* = 0.5 V. Although the performance of the fabricated mHEMT is not comparable to that of the In_0.53_Ga_0.47_As/InAs/In_0.53_Ga_0.47_As composite channel HEMT on an InP substrate, our fabricated mHEMT shows outstanding high-frequency characteristics compared to a single InGaAs channel HEMT on an InP substrate and other mHEMT structures because of the excellent transport properties of the composite channel on a GaAs substrate. Additionally, our fabricated device is operated at a *V_DS_* = 0.5 V, which has a lower power consumption than other group devices’ operational voltage. These excellent performances are mainly attributed to the well-grown In_0.53_Ga_0.47_As/InAs/In_0.53_Ga_0.47_As composite channel structure by using an In_0.52_AlAs buffer layer on a GaAs substrate, and a fabricated mHEMT would be a good candidate for the high-frequency device in both 5G and 6G communications through further scaling-down of device feature size.

## 4. Conclusions

The 100 nm In_0.53_Ga_0.47_As/InAs/In_0.53_Ga_0.47_As composite channel metamorphic high electron mobility transistors (mHEMTs) on a GaAs substrate exhibited excellent logic characteristics as well as high-frequency RF performances. These outstanding performances are due to the excellent carrier transport properties of the well-grown In_0.53_Ga_0.47_As/InAs/In_0.53_Ga_0.47_As composite channel mHEMT structure on a GaAs substrate and an optimized fabrication process. The proposed mHEMT structure on a GaAs substrate, together with optimized source/drain and gate technologies, will potentially improve logic and high-frequency characteristics. Furthermore, the proposed mHEMT structure grown on a large-size GaAs substrate could be indispensable for large-volume manufacturing.

## Figures and Tables

**Table 1:** Extracted intrinsic small-signal parameters.

Intrinsic Parameters	Measured *f_T_*	Modeling *f_T_*
*g_mi_* = 2.0 mS/µm	261 GHz	258 GHz
*g_ds_* = 0.22 mS/µm		
*R_g_* = 70 Ω-µm		
*R_s_* = 360 Ω-µm		
*R_d_* = 360 Ω-µm	**Measured *f_max_***	**Modeling *f_max_***
*R_i_* = 100 Ω-µm	304 GHz	309 GHz
*C_gs_* = 0.65 fF/µm		

**Table 2:** Performance parameters of the pHEMTs and mHEMTs with a *L_g_* of 100 nm.

	[20]	[18]	[21]	[22]	This Work
Substrate	InP	InP	GaAs	GaAs	GaAs
Channel	In_0.68_Ga_0.32_As	In_0.53_Ga_0.47_As /InAs/ In_0.53_Ga_0.47_As	In_0.65_Ga_0.35_As/ In_0.53_Ga_0.47_As	In_0.6_Ga_0.4_As	In_0.53_Ga_0.47_As /InAs/ In_0.53_Ga_0.47_As
Buffer layer	InAlAs buffer	InAlAs buffer	Linear In_x_Al_0.48_Ga_0.52-x_As	Graded InAlAs	500 nm In_0.52_AlAs
*L_g_* [nm]	100	100	100	100	100
*f_T_* [GHz]	183	421	220	210	261
*f_max_* [GHz]	230	620	300	252	304
*L_g_f_T_* [GHz-µm]	18.3	42.1	22.0	21.0	26.1
*V_DS_* [V]	0.5	0.7	1	1	0.5
Passivation	100 nm SiN_x_	-	250 nm SiN_x_	50 nm SiN_x_	-
